# From omics and etics to policy and ethics: regulating evolution

**DOI:** 10.3389/fgene.2013.00172

**Published:** 2013-09-19

**Authors:** Nils Hoppe

**Affiliations:** Centre for Ethics and Law in the Life Sciences, Leibniz Universität HannoverHannover, Germany

**Keywords:** genomics and genetics, ethics, collaborative research, lawyers, sociology

Few technologies have experienced such an explosive development and significant increase in utility as the study, description and manipulation of the genes that spell out many of the functions of human life—it almost seems as if the science of our genes is autopoietic in terms of generating innovation. Genetic information is used to address large-scale problems, for example where whole genome and exome sequencing advances the understanding of rare diseases. It is used to tailor individualised health interventions, such as where the identification of a HER2 mutation may open the therapeutic pathway to a treatment with Herceptin or where evidence of a BRCA mutation may trigger potentionally life-saving surgery. From Sir Alec Jeffrey's discovery of DNA similarities, used to identify perpetrators of crime and family relations in immigration cases, to direct-to-consumer genetic testing, used to identify a child's father (or possibly more frequently—establishing the fact that the putative father is not the genetic father) and an individual's genetic disease probability (relative to some nebulous population), genetics and -omics are becoming all-pervasive and, above all, affordable. The cost of whole genome sequencing has decreased dramatically in little more than 10 years—Figure 1 (Wetterstrand, 2013) shows the decreasing cost of sequencing a genome at NHGRI: from just under $100 m in 2001 to just over $5000 in 2013. 23 and Me, a prominent provider of direct-to-consumer genetic testing for disease probability and ancestry, started in 2007 at a cost of $999 per test and now only charges $99 for its service.^1^ This is the mainstreaming of high-impact technologies: similar to the development and impact of the internet—once available to the specialised privileged few, with limited features and at great expense only—the genetic revolution has taken powerful information out of the hands of the few and made it available to many. This increase in density and availability of meaningful information leads to a perceivable public trend in seeing privacy merely as a symptom of an informational deficiency, i.e., there is a plausible argument that the perceived explosion in information sharing online may well lead to a desensitization, rather than an increased fear for privacy. At the same time, regulatory sciences and the disciplines looking at ethical, legal, and social implications of this type of technology doggedly adhere to their time-honored, heterogeneous and siloed methodological traditions: bar some notable exceptions, lawyers speak to lawyers, ethicists speak to ethicists, sociologists speak to sociologists. Where debate transcends disciplinary boundaries, it is often received with suspicion of the other discipline's (or country's) scholarly conventions, language, and style.

**Figure 1:**
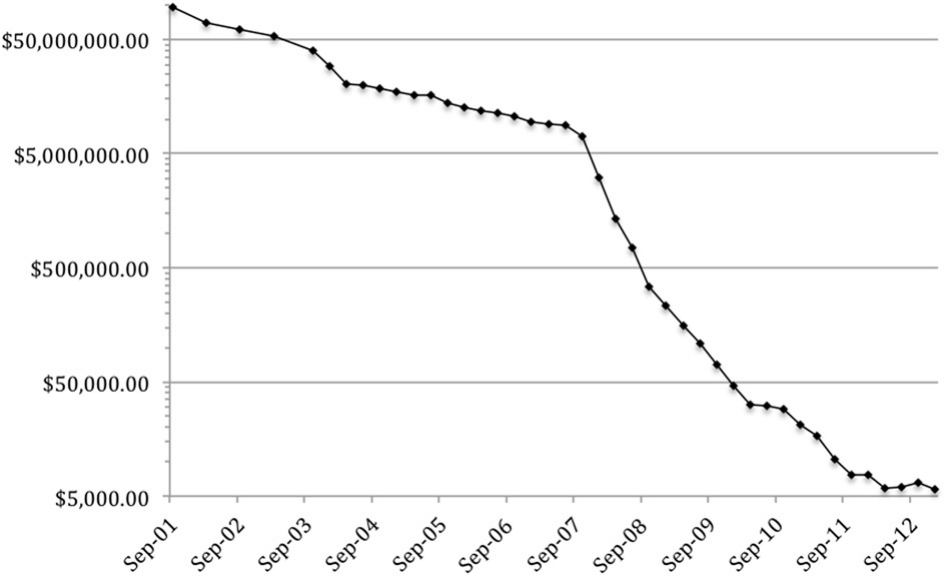
**DNA sequencing costs: data from the NHGRI genome sequencing program**.

This shows, amongst other things, that we have still not achieved a significant level of genuine interdisciplinary, transdisciplinary and international collaboration to address the significant ELSI issues that have arisen in the past years. So where does this leave policy and ethics? The creep of these groundbreaking technologies into every aspect of modern medicine and society has a profound impact on social cohesion and human self-perception. Issues of genetic discrimination and stigmatisation have been the subject of extensive medico-legal and ethical discussion for very many years. If vast portions of our selves are generated using a simple, now legible, alphabet, what does this mean for the crude distinctions societies have made on the basis of phenotype for so long? Will this lead to a shift from phenotype discrimination to genotype discrimination or to the enlightened view that we are all “made of the same stuff”?

The speed and significance of the societal impact of -omics and -etics, for which we already have the evidence-base, needs to be matched by coherent and efficient research (infra)structures addressing ethical, legal and social implications of these technologies. Moreover, the output of these ELSI activities needs to be viewed as the engine driving a translational process—this output needs to inform policy level decision-making just as fast as the latest identification of a biomarker may lead to a bedside benefit. 2012 saw the launch of a global initiative seeking to give the necessary impulse to create such a structure and a plethora of endeavours to fill this proposal with life are under way (Kaye et al., 2012). The idea is this: the matching scholarly and policy response to developments in genetics and genomics is to undertake high-throughput, massively-open, global-scale, next-generation ELSI work. All are welcome.

## Footnotes

^1^http://mediacenter.23andme.com/press-releases/23andme-lowers-price-again-ends-subscriptions/. Accessed 25 May 2013.
